# EFFECT OF DIURNAL INTERMITTENT FASTING (DIF) ON ANTIOXIDANT AND PRO INFLAMMATORY MEDIATORS ACTIVITY IN MALE RAT MODEL OF TYPE 2 DIABETES MELLITUS

**DOI:** 10.21010/Ajidv19i2S.7

**Published:** 2025-10-17

**Authors:** Selamat Ginting, Chrismis Novalinda Ginting, Ok Yulizal, Jekson Martiar Siahaan

**Affiliations:** 1 Doctoral Program, Universitas Prima Indonesia, Jalan Sampul No. 3, Medan, Indonesia; 2 Department of Nursing, Institut Kesehatan Deli Husada Deli Tua, Sumatera Utara, Indonesia; 3 Department of Physiology, Faculty of Medicine, Institut Kesehatan Deli Husada Deli Tua, Sumatera Utara, Indonesia

**Keywords:** Diurnal intermittent fasting, type 2 diabetes mellitus, oxidative stress, interleukin-6, superoxide dismutase, inflammation

## Abstract

**Background:**

Type 2 diabetes mellitus (T2DM) is a chronic metabolic disorder characterized by persistent hyperglycemia, oxidative stress, and systemic inflammation. Diurnal intermittent fasting (DIF), a fasting pattern synchronized with circadian rhythms, has been proposed as a potential strategy to alleviate metabolic disturbances, but evidence from controlled animal studies remains limited.

**Materials and Methods:**

This experimental study employed a post-test-only control group design using thirty-six male Wistar rats. T2DM was induced by streptozotocin (65 mg/kg) and nicotinamide (230 mg/kg). Animals were randomized into four groups: diabetic control (G1), and three DIF-treated groups fasting two (G2), three (G3), and six (G4) days per week. Blood glucose was measured weekly. On day 28, serum levels of superoxide dismutase (SOD) and interleukin-6 (IL-6) were analyzed using enzyme-linked immunosorbent assay (ELISA).

**Results:**

DIF significantly reduced blood glucose levels in all intervention groups compared to the control (p < 0.05). The G4 group showed the highest SOD activity and the greatest IL-6 reduction (p < 0.05). However, there was no significant glucose difference between G3 and G4, suggesting a plateau in glycemic improvement.

**Conclusion:**

DIF improves glycemic control, enhances antioxidant defense through increased SOD activity, and reduces systemic inflammation via IL-6 suppression in a T2DM rat model. These findings support the potential of DIF as a complementary therapeutic approach for T2DM, although further research is needed to determine the optimal fasting regimen and its applicability in humans.

## Introduction

Type 2 diabetes mellitus (T2DM) continues to rise as a major public health concern across the globe, significantly reducing life expectancy and increasing the strain on healthcare systems (Khan *et al.*, 2020; Huang et al., 2022; Manurung *et al.*, 2025). It is currently ranked among the top ten causes of mortality worldwide, with more than one million deaths recorded annually (Khan *et al.*, 2020). Although developed countries show a high prevalence, Southeast Asian nations—including Indonesia, Malaysia, Thailand, and Vietnam—have experienced a marked increase in T2DM incidence over the past two decades (Abdelrahim *et al.*, 2020).

T2DM is a chronic metabolic condition defined by persistent hyperglycemia, which arises from both insulin resistance and β-cell dysfunction (Siahaan *et al.*, 2021; Situmorang *et al.*, 2024). These metabolic disturbances promote overproduction of reactive oxygen species (ROS), triggering oxidative stress and chronic inflammation—two central mechanisms that underlie diabetes-related complications. Superoxide dismutase (SOD) plays a key protective role by converting harmful superoxide radicals into less reactive molecules, but its activity is often reduced in diabetic states, allowing ROS to accumulate and damage tissues (Siahaan et al., 2020). Meanwhile, hyperglycemia also induces the release of inflammatory mediators such as interleukin-6 (IL-6), which worsens insulin resistance and contributes to systemic inflammation (Retta *et al.*, 2025).

Intermittent fasting (IF), which alternates between periods of eating and fasting, has gained popularity for its beneficial effects on metabolic regulation. Evidence suggests that IF can lower blood glucose levels, improve insulin sensitivity, and reduce oxidative stress and inflammatory cytokines (Welton *et al.*, 2020; Vasim et al., 2022). Experimental studies have shown that IF enhances endogenous antioxidant responses, including increased SOD activity, and reduces inflammation through downregulation of IL-6 (Faris *et al.*, 2020; Vasim *et al.*, 2022).

Among the different IF patterns, diurnal intermittent fasting (DIF)—which restricts food intake from dawn until sunset—is increasingly recognized for its alignment with circadian biology, potentially optimizing metabolic responses. DIF is commonly practiced in religious or cultural settings and has been associated with improved glycemic control and reductions in oxidative stress markers (Manoogian *et al.*, 2021; Acosta-Rodríguez *et al.*, 2022; Madkour *et al.*, 2022; Bajaj and Kaur, 2023). Despite growing interest, limited studies have examined how different frequencies of DIF affect oxidative stress and inflammatory markers in controlled diabetic models. Understanding these effects may provide insight into DIF’s role as a supportive strategy in diabetes management (Nowosad and Sujka, 2021; Chadwick, 2024). **Therefore, this study investigated how varying the frequency of DIF influences serum levels of SOD and IL-6 in male Wistar rats with T2DM induced by streptozotocin and nicotinamide.** The outcomes are expected to contribute valuable preclinical data supporting the therapeutic potential of DIF in modulating oxidative and inflammatory pathways in diabetes.

## Materials and Methods

### Research Design

This experimental study employed a post-test-only control group design to assess the effects of varying frequencies of diurnal intermittent fasting (DIF) on oxidative stress and inflammation in a rat model of type 2 diabetes mellitus (T2DM). A total of 36 male Wistar rats (8 weeks old; 200–250 g) were randomly allocated into four groups (n = 9 per group):

- **G1 (Control):** No fasting, standard diet and water *ad libitum*
- **G2:** DIF twice weekly (Monday, Saturday)
- **G3:** DIF three times weekly (Tuesday, Thursday, Saturday)
- **G4:** DIF six times weekly (Monday to Saturday)

The use of male rats was intended to reduce hormonal variability, particularly fluctuations in estrogen and progesterone, which could confound metabolic and inflammatory outcomes in female subjects. This approach is consistent with widely accepted practices in preclinical metabolic research.

All fasting groups went through a 14-hour fasting period daily (5:00 AM–7:00 PM), during which food was withheld while water remained freely available. Non-fasting periods allowed unrestricted access to standard rodent chow.

T2DM was induced using a combination of intravenous streptozotocin (STZ; 65 mg/kg body weight) and intraperitoneal nicotinamide (NA; 230 mg/kg), following a two-week acclimatization phase. Rats with fasting blood glucose levels ≥126 mg/dL, measured three days post-induction, were considered diabetic and included in the intervention phase.

### Data Collection Procedures

Animals were housed under standardized laboratory conditions: temperature 22–24 °C, humidity 40–60%, and a 14:10 light-dark cycle (lights on from 5:00 AM to 7:00 PM) aligned with fasting schedules. Each polycarbonate cage housed no more than three animals and was equipped with sterilized wood-chip bedding and environmental enrichment (e.g., wooden blocks) to reduce stress and promote natural behavior. Bedding was replaced every 2–3 days.

Blood glucose levels were monitored weekly (days 7, 14, 21, and 28) via tail-tip sampling using a glucometer. On day 28, terminal blood collection was performed under brief inhalation anesthesia (isoflurane, 2%) via retro-orbital sinus puncture. Serum was isolated by centrifugation (3000 rpm, 15 min, 4 °C) and stored at –80 °C until analysis. Levels of interleukin-6 (IL-6) and superoxide dismutase (SOD) were measured using ELISA kits specific for rats (Abbkine, USA), following the manufacturer’s protocols. Optical densities were read at 450 nm, and concentrations were derived from standard calibration curves.

All procedures were conducted by trained personnel, in accordance with institutional and international ethical guidelines. Humane endpoints were strictly observed, and animals were euthanized using isoflurane overdose followed by cervical dislocation to ensure a painless and ethical termination. This study received ethical clearance from the Ethics Committee of Universitas Sumatera Utara (No. 0571/KEPH-FMIPA/2023), and all protocols complied with the ARRIVE guidelines and the principles of the 3Rs (Replacement, Reduction, Refinement).

## Data Analysis

Statistical analysis was performed using GraphPad Prism version 10 (GraphPad Software, USA). Data distribution was assessed using the Shapiro–Wilk test. One-way ANOVA was applied to detect differences among groups, followed by Tukey’s post hoc test for pairwise comparisons. Statistical significance was set at p < 0.05. Results are presented as mean ± standard deviation (SD).

## Results

### Blood Glucose Levels Before and After STZ-NA Induction

**Figure 1:**
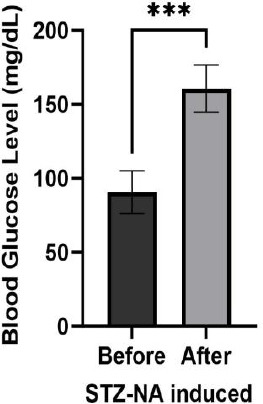
Effect of STZ-NA induction on fasting blood glucose levels (***p < 0.05)

Following administration of streptozotocin (STZ; 65 mg/kg BW) and nicotinamide (NA; 230 mg/kg BW), a significant elevation in fasting blood glucose levels was observed across all groups compared to pre-induction values (p < 0.05), as shown in Figure 1. The post-induction mean values exceeded 126 mg/dL, confirming successful establishment of the T2DM model. No mortality was recorded during the induction phase or throughout the entire experimental period. All animals remained viable and clinically stable.

### Effect of Diurnal Intermittent Fasting (DIF) on Blood Glucose Levels

**Figure 2:**
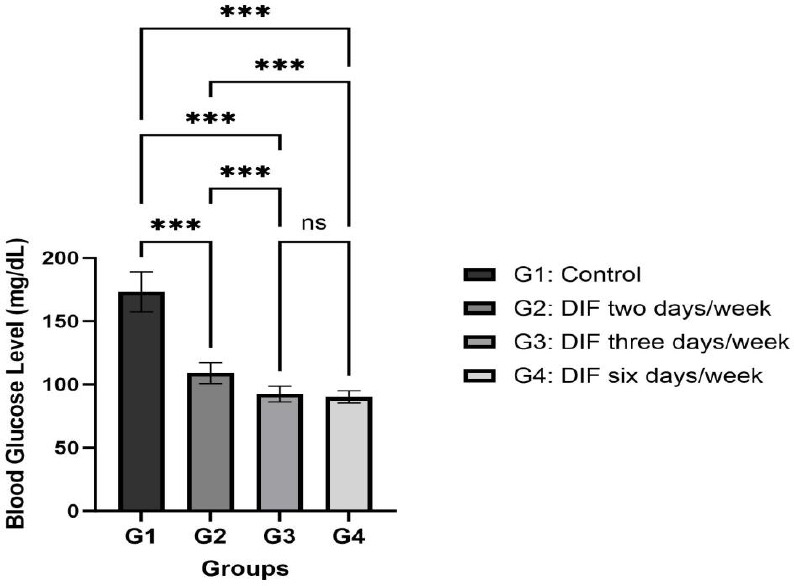
Effects of different frequencies of DIF on blood glucose levels in T2DM rats (***p < 0.05; ns: not significant)

Figure 2 shows that DIF intervention significantly lowered fasting blood glucose levels in all treated groups (G2–G4) compared to the diabetic control (G1) (p < 0.05, one-way ANOVA followed by Tukey’s test). Notably, while both G3 (three days/week) and G4 (six days/week) exhibited marked reductions, the difference between them was not statistically significant (ns), suggesting a plateau effect beyond three fasting days per week.

### Effect of DIF on Superoxide Dismutase (SOD) Activity

**Figure 3:**
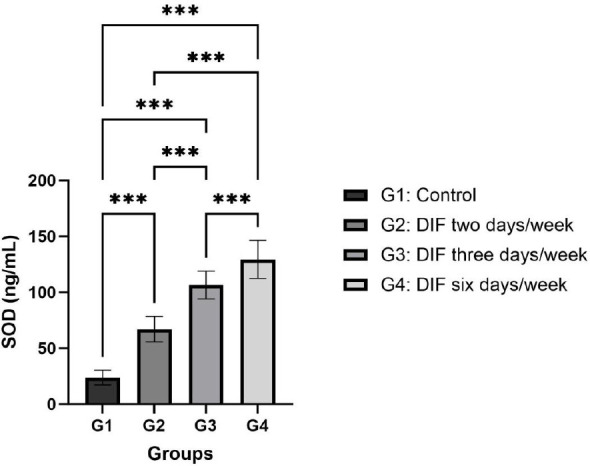
SOD levels in T2DM rats following DIF intervention (***p < 0.05)

As illustrated in Figure 3, DIF significantly enhanced SOD activity in all intervention groups relative to the diabetic control (p < 0.05). The increase was frequency-dependent, with the G4 group (six days/week) demonstrating significantly higher SOD levels compared to G2 and G3. These findings suggest a progressive improvement in endogenous antioxidant defense with increased fasting frequency.

### Effect of DIF on Serum Interleukin-6 (IL-6) Levels

**Figure 4:**
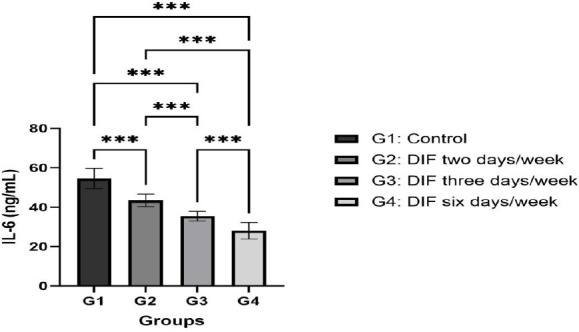
Serum IL-6 concentrations in T2DM rats after DIF intervention (***p < 0.05)

DIF also led to a significant reduction in IL-6 levels across all treatment groups compared to the diabetic control (G1) (p < 0.05), as shown in Figure 4. The group subjected to six-day fasting (G4) showed the lowest IL-6 levels, significantly different from both G2 and G3, indicating that higher DIF frequency may exert stronger anti-inflammatory effects.

## Discussion

The mechanism of STZ toxicity is well-documented, involving DNA alkylation—primarily via its methyl-nitrosourea moiety—which induces strand breaks, poly (ADP-ribose) polymerase (PARP) activation, and subsequent NAD^+^/ATP depletion leading to pancreatic β-cell necrosis (Ajao *et al.*, 2023; Sinaga *et al.*, 2025). Additionally, STZ induces reactive oxygen species (ROS) generation, leading to mitochondrial dysfunction, apoptosis, and ATP depletion (Rais *et al.*, 2022). These ROS further stimulate nitric oxide (NO) production, exacerbating DNA damage and disrupting the Krebs cycle (Pappas, 2023) Nicotinamide (NA), on the other hand, acts as a protective agent by replenishing NAD^+^ levels and mitigating oxidative stress (Covarrubias *et al.*, 2021). The combination of STZ and NA produces sustained hyperglycemia that closely mimics human type 2 diabetes mellitus (T2DM), making it a robust experimental model for evaluating therapeutic interventions like diurnal intermittent fasting (Yan *et al.*, 2022). In this study, DIF significantly reduced blood glucose levels in all fasting groups (G2, G3, and G4) compared to the T2DM control group (G1). The reduction in blood glucose levels following DIF intervention is likely attributable to a combination of suppressed insulin secretion during fasting periods and metabolic adaptations that enhance insulin sensitivity (Albosta and Bakke, 2021). One proposed mechanism involves increased glucose uptake by peripheral tissues and improved glucose homeostasis through inhibition of excessive lipogenesis and suppression of hepatic gluconeogenesis (Mishra and Singh, 2020; Vasim *et al.*, 2022; Shah and Wondisford, 2023). Additionally, DIF may trigger a “metabolic switch,” shifting the body’s primary energy source from glucose to lipids, thus contributing to more effective and sustained reductions in glycemia (Liu *et al.*, 2025). These findings align with evidence that intermittent fasting strategies can significantly improve glycemic control and reduce the risk of chronic diabetic complications (Vasim *et al.*, 2022).

However, it is interesting to note that this study also found no significant difference in blood glucose levels between the three-days (G3) and six-days (G4) per-week fasting groups. This phenomenon indicates a “plateau effect” in the metabolic benefits obtained from increasing the frequency of DIF fasting. Several physiological mechanisms can explain this plateau effect. First, metabolic adaptations such as increased insulin sensitivity and enhanced glucose utilization may reach a plateau, beyond which further benefits from increased fasting frequency become minimal (de Cabo and Mattson, 2019). The increase involves upregulation of glucose transporters—particularly GLUT4—in skeletal muscle and adipose tissue, facilitating maximal glucose uptake during repeated fasting conditions (Chadt and Al-Hasani, 2020). Second, prolonged fasting elicits hormonal compensatory responses: elevated glucagon, cortisol, and catecholamine levels act as homeostatic mechanisms, promoting hepatic glucose release that may counterbalance the glucose-lowering effects of fasting (Karimi *et al.*, 2020). Third, the liver has a limited capacity to suppress gluconeogenesis and glycogenolysis during prolonged or repeated fasts, as hepatic glucose production becomes resistant to further hormonal control beyond certain metabolic thresholds (Chadt and Al-Hasani, 2020; Zhou *et al.*, 2024). After reaching a certain point of metabolic adaptation, increasing fasting frequency may no longer yield further improvements in blood glucose regulation, as processes such as insulin sensitivity and glucose utilization efficiency approach their maximal capacity (Hall, 2024). Thus, these findings suggest that although DIF is effective in improving glycemic control in T2DM models, there is a biological threshold at which increasing fasting frequency no longer results in significant additional glycemic improvements (Wildes *et al.*, 2024; Zheng *et al.*, 2024).

The significant increase in SOD activity observed in the six-days-per-week fasting group (p < 0.05) suggests that DIF is effective in reducing oxidative stress in the T2DM model. SOD plays a crucial role in neutralizing superoxide radicals, thereby maintaining redox balance and preventing oxidative damage to cells and tissues (Palma *et al.,*. 2020). In addition, DIF also resulted in a significant reduction in IL-6 levels, further supporting the notion that DIF reduces systemic inflammation in T2DM (p < 0.05). IL-6 is a key inflammatory mediator in insulin resistance and vascular damage associated with chronic inflammation. (Bowker *et al.*, 2020). Our results indicate that diurnal intermittent fasting (DIF) not only effectively lowers blood glucose but also significantly enhances antioxidant defense - evidenced by elevated SOD activity - and reduces inflammation, as shown by decreased IL-6 levels. These outcomes underscore DIF’s promise as a therapeutic intervention for managing oxidative stress and inflammation in T2DM. Although our study did not directly explore the molecular pathways underlying these effects, the findings are consistent with existing literature showing that fasting activates signaling cascades responsible for boosting redox balance and attenuating inflammatory responses (Joaquim *et a*l., 2022; Marko *et al.*, 2024). DIF likely creates a cellular environment that simultaneously supports antioxidant capacity and suppresses pro-inflammatory cytokines. Nevertheless, further studies employing molecular analyses—such as measuring AMPK/mTOR activity, autophagy markers, or the Nrf2 antioxidant pathway—are essential to clarify the precise mechanisms at play (Nieto *et al.*, 2025).

Although our preclinical findings indicate that DIF markedly enhances glycemic control, bolsters antioxidant capacity, and reduces systemic inflammation in a T2DM model, translating these benefits to human populations warrants caution. In humans, insufficient macro- and micronutrient intake during eating windows may lead to nutritional deficiencies, negatively affecting metabolic and immune functions (Haileselassie *et al.*, 2022). Individuals taking antidiabetic medications are at heightened risk of hypoglycemia during fasting, a concern further compounded by possible dehydration from reduced fluid intake (Grajower and Horne, 2019). Moreover, adhering to strict fasting regimens can be challenging; cultural norms, social commitments, and economic constraints significantly influence compliance ( Trabelsi *et al.*, 2022; Arslan and Aydın, 2024).

Furthermore, physiological, metabolic, and hormonal differences between mice and humans are critical factors that must be considered in designing further clinical studies (Pandey and Dvorakova, 2020). To confirm the safety and efficacy and determine the optimal frequency and duration of fasting in humans, rigorous clinical studies are needed, especially through randomized controlled clinical trials (RCTs) and long-term observational studies (Albosta and Bakke, 2021). As a practical implication, further clinical studies need to explore additional parameters such as changes in lipid profiles, body mass index (BMI), and other biomarkers of inflammation and oxidative stress. In addition, it is important to evaluate clinical factors that may influence an individual’s response to DIF, including disease duration, use of certain medications, nutritional status, and genetic factors (Moon *et al.*, 2020). These studies will help to design more personalized interventions and increase the likelihood of successful implementation of DIF in daily clinical practice.

Thus, although the results from animal models provide a strong scientific basis for the potential benefits of DIF, caution is needed before its widespread application to human patients with T2DM. Further clinical research is necessary to bridge the gap between findings from animal models and their application in humans. Randomized controlled clinical trials, long-term observational studies, and molecular mechanistic studies to understand variations in response by demographics and health status will be essential components to determine whether the benefits seen in animal models can truly translate into safe and effective intervention strategies in human populations.

## Conclusions

In conclusion, our study demonstrates that DIF significantly reduces blood glucose levels, enhances SOD levels, and lowers IL-6 levels in a rat model of T2DM. The group fasting six-days-per-week had the most pronounced effects. However, no significant difference in glucose reduction was found between the three-days and six-days-per-week DIF intervention, suggesting a plateau in glycemic improvement beyond a certain fasting frequency.

Based on these results, we recommend further research to determine the optimal fasting frequency and duration for maximum metabolic benefit. Future studies should also focus on validating these findings in human clinical trials to assess safety and efficacy and explore the underlying molecular mechanisms and potential effects on additional metabolic and inflammatory markers.

### Conflict of interests:

The authors declare that there is no conflict of interest associated with this study.

**List of Abbreviations:**
T2DM: Type 2 diabetes mellitus;
SOD: superoxide dismutase;
ELISA: enzyme-linked immunosorbent assay;
IL-6: interleukin-6;
ROS: reactive oxygen species;
DIF: diurnal intermittent fasting;
STZ: streptozotocin
NA: nicotinamide
